# Recent progress in oxynitride photocatalysts for visible-light-driven water splitting

**DOI:** 10.1088/1468-6996/16/3/033506

**Published:** 2015-05-26

**Authors:** Tsuyoshi Takata, Chengsi Pan, Kazunari Domen

**Affiliations:** 1 Global Research Center for Environment and Energy based on Nanomaterials Science (GREEN), National Institute for Materials Science (NIMS), 1-1 Namiki, Tsukuba-city, Ibaraki 305-0044, Japan; 2 Department of Chemical System Engineering, School of Engineering, The University of Tokyo, 7-3-1 Hongo, Bunkyo-ku 113-8656, Japan

**Keywords:** photocatalyst, water splitting, semiconductor, cocatalyst, visible light, oxynitride

## Abstract

Photocatalytic water splitting into hydrogen and oxygen is a method to directly convert light energy into storable chemical energy, and has received considerable attention for use in large-scale solar energy utilization. Particulate semiconductors are generally used as photocatalysts, and semiconductor properties such as bandgap, band positions, and photocarrier mobility can heavily impact photocatalytic performance. The design of active photocatalysts has been performed with the consideration of such semiconductor properties. Photocatalysts have a catalytic aspect in addition to a semiconductor one. The ability to control surface redox reactions in order to efficiently produce targeted reactants is also important for photocatalysts. Over the past few decades, various photocatalysts for water splitting have been developed, and a recent main concern has been the development of visible-light sensitive photocatalysts for water splitting. This review introduces the study of water-splitting photocatalysts, with a focus on recent progress in visible-light induced overall water splitting on oxynitride photocatalysts. Various strategies for designing efficient photocatalysts for water splitting are also discussed herein.

## 1. Introduction

Efficient utilization of solar energy is one of the most important issues for the realization of a green, sustainable society. The conversion of solar energy into useful forms that are suitable for storage and transportation is required. Water splitting into hydrogen and oxygen using light energy, which corresponds to the light reaction in photosynthesis on chlorophyll, could be the most fundamental and simplest process for solar energy utilization. When this process can be efficiently driven by solar irradiation, it is possible to produce clean and renewable hydrogen fuel. The obtained hydrogen would be usable for catalytic fixation of CO_2_ or N_2_ to produce hydrocarbon/alcohol or ammonia as fuels and chemicals. Of course, direct conversion of hydrogen to electricity by a fuel cell is also possible.

**Figure 1.**
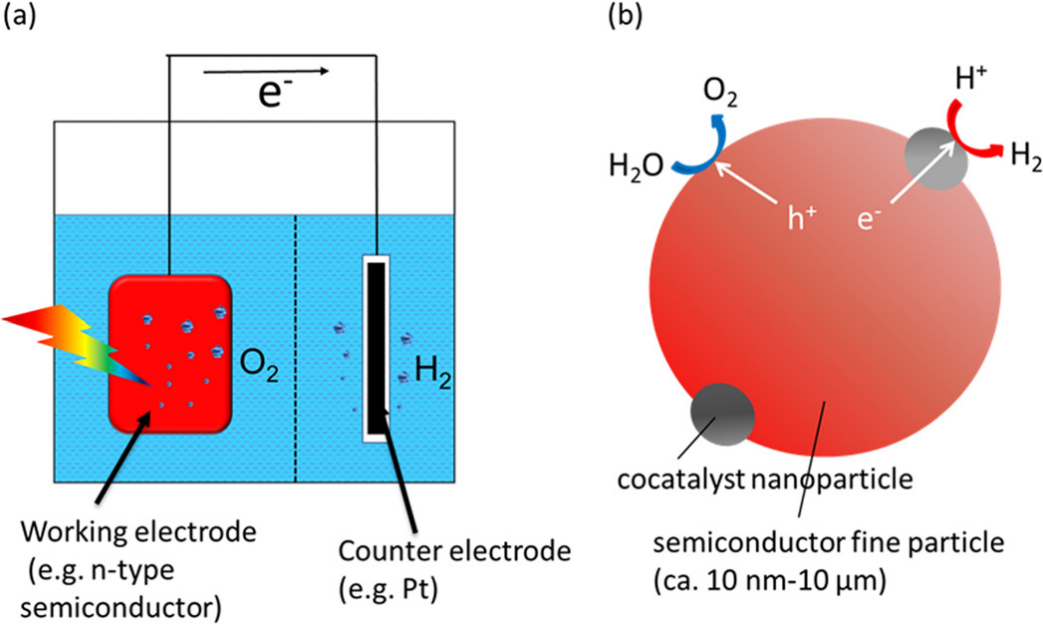
Schematic models of (a) photoelectrochemical and (b) photocatalytic water splitting.

Since the discovery of the Honda–Fujishima effect [1], in which photoelectrochemical water splitting by bandgap excitation on single-crystal TiO_2_ (rutile) was demonstrated, photolysis of water using semiconductors has been studied with considerable attention. The Honda–Fujishima effect was demonstrated using an electrochemical method based on a semiconductor electrode and a counter electrode assisted by a bias voltage. A basic construction of a photoelectrochemical water splitting system is depicted in figure 1(a). Another prevailing method has been photocatalysis using a micrometer-scale particulate semiconductor, as depicted in figure 1(b), which is similar to a miniaturized photoelectrochemical system. This method is considered to enable enhanced reaction efficiency due to the larger available surface area, and is extensible to a very large scale because of its simplicity [2].

In the early stages of this research, stoichiometric photocatalytic water splitting into H_2_ and O_2_ was not as easy as was theoretically expected because of various obstacles, concurrent reverse reaction, instability of photocatalyst, poor variation in the selection of photocatalyst and cocatalyst materials, etc. However, remarkable progress has been made, and many difficulties and problems have been overcome. In fact, overall water splitting with a high reaction efficiency on many UV light-sensitive photocatalysts had been achieved until a decade ago [3–5], and recently, photocatalysts have been developed that are capable of overall water splitting under visible-light irradiation [5–7]. The present review introduces the progress in the study of photocatalysts for overall water splitting, with a focus on recent advances in visible-light water splitting on oxynitride photocatalysts. Representative topics in this overview are introduced in the following sections.

## 2. Theory of photocatalytic water splitting

**Figure 2.**
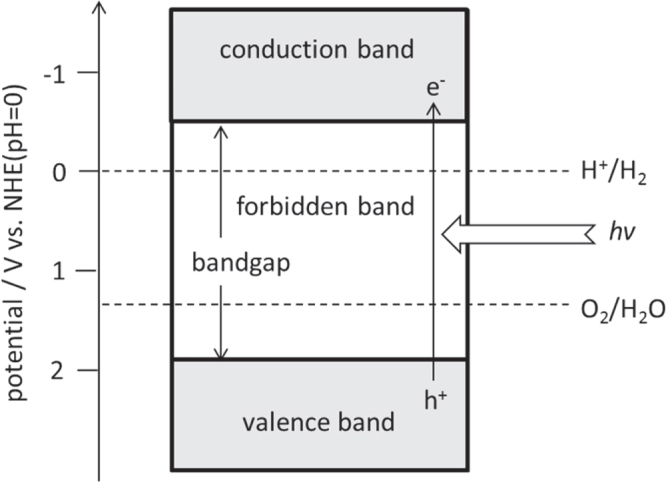
Basic principles of water splitting on a semiconductor photocatalyst.

The basic principle of photocatalytic water splitting is shown in figure 2. Generally, inorganic-based semiconductors are used for photocatalysis. Solid materials have an electronic structure with some empty and some filled electronic states. If there is an energy gap between these two sets of electronic states, the material is classified as a semiconductor or insulator. The filled and empty energy states and the energy gap are called the valence band, the conduction band, and the bandgap, respectively.

Electrons are excited from the valence band to the conduction band by absorbing photons with energies exceeding the bandgap energy of the semiconductor, generating photoexcited electrons and positive holes in the conduction and valence bands, respectively. These charged species then diffuse and reach the surface of the photocatalyst particle, where they subsequently participate in redox reactions, as denoted in equations (1) and (2), and summarized in equation (3)

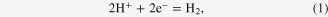


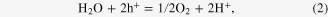


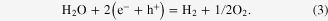


One prerequisite for photocatalytic water splitting is that the conduction band minimum (CBM) and valence band maximum (VBM) for a semiconductor material should straddle the redox potentials for H^+^/H_2_ and O_2_/H_2_O. Thermodynamically, the theoretical minimum photon energy required to split water via a 4-electron transfer step is 1.23 eV. This photon energy corresponds to a 1000 nm wavelength. However, in practice, a somewhat higher photon energy is necessary due to the presence of overpotentials, and a photon energy of 1.7 eV (730 nm) is thought to be a realistic goal. It should be noted that the bandgap of a photocatalyst usable for water splitting is too large (>1.23 eV) for it to be categorized as a semiconductor, and such materials should be correctly categorized as insulators. However, it has become common practice to nevertheless refer to these materials as semiconductors, and we will therefore continue do so herein.

Another requirement is that the photocatalyst should have a suitably small particle size, so that the photogenerated charges can reach surface reaction sites by diffusion. The reaction rate of water splitting is primarily determined by the relative rates between charge-recombination and -injection to the reactant molecules. Empirically, the particle size for photocatalysts usable in common experiments is approximately 10 nm to 10 *μ*m. However, an excessively small particle size is disadvantageous since a sufficient amount of light absorption can not be achieved.

**Figure 3.**
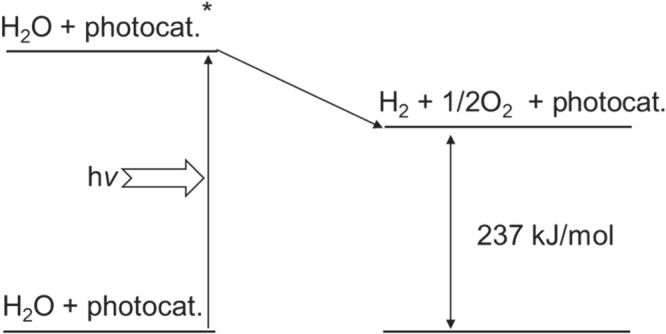
Energy diagram of photocatalytic water splitting.

In photocatalysis, the semiconductor properties of photo-absorption and charge generation are important, but so is the catalytic functionality. To efficiently perform surface redox reactions, a semiconductor photocatalyst is usually combined with a cocatalyst. The cocatalyst corresponds to the counter electrode of a photoelectrochemical water splitting system. Therefore, the components which tend to be used as efficient electrocatalysts for H^+^-reduction with low overpotentials, such as Pt, Rh, Ru and Ni are also employed as active cocatalysts in photocatalysis [8–11]. However, one difference should be taken into consideration for the design of an efficient water splitting photocatalyst; that is, the difference in scales between photocatalysis and photoelectrolysis. In photocatalysis, reduction and oxidation sites are much closer than in a photoelectrochemical system. Therefore, H_2_ and O_2_ produced on a photocatalyst particle have a possibility to recombine, regenerating water. Water formation from H_2_ and O_2_ is a downhill reaction, with a decrease in Gibbs free energy of 237 kJ mol^−1^, and is thermodynamically favorable, as diagramed in figure 3. If a suitable cocatalyst for the promotion of water formation coexists with a photocatalyst, the reaction promptly proceeds spontaneously. Indeed, most active H_2_ evolution cocatalysts are also highly active for water formation. In this case, the water splitting reaction does not proceed efficiently. Therefore, careful design and structural control are needed to prevent the reverse reaction on micrometer-scale photocatalyst particles. For a cocatalyst to promote water splitting, a dual functionality is required; the cocatalyst must promote H_2_ and/or O_2_ evolution while preventing the unfavorable reverse reaction to regenerate water. Eventually, water splitting photocatalysts should be developed with consideration of all three aforementioned requirements.

## 3. Measurement of photocatalytic activity for water splitting

**Figure 4.**
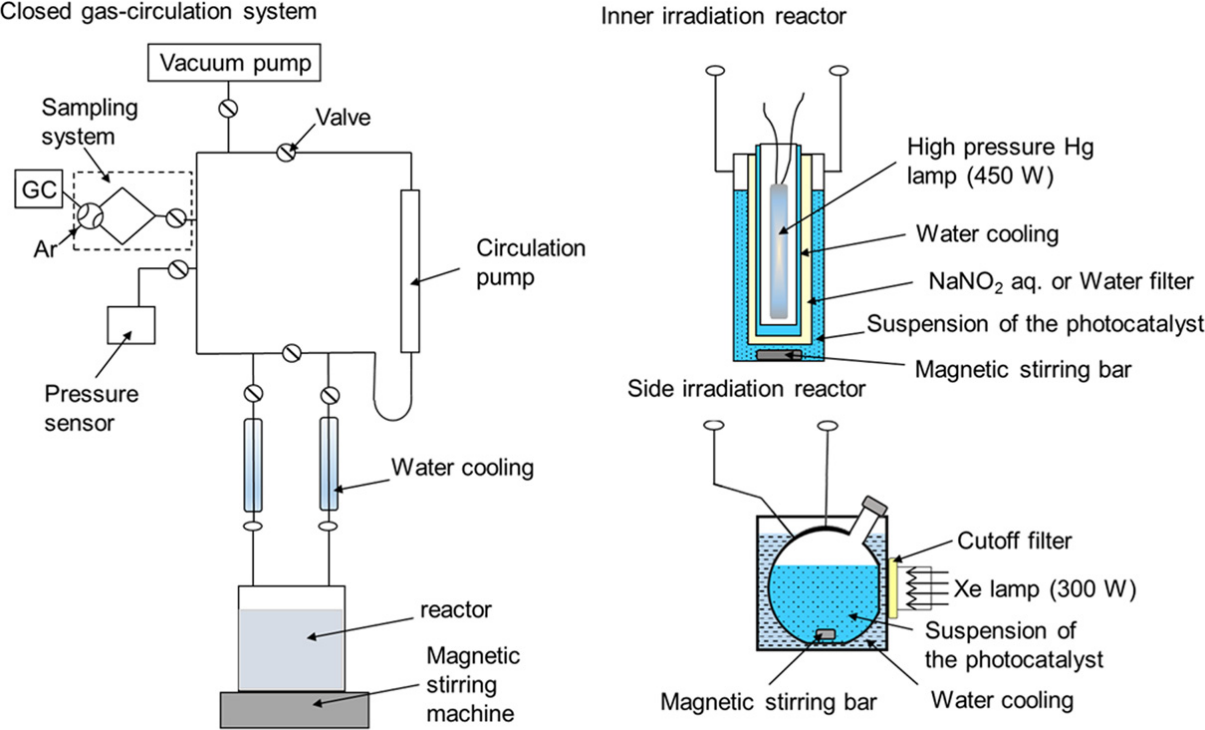
Schematic of the apparatus for photocatalytic water splitting.

Photocatalytic water splitting reactions are usually carried out in a closed gas-circulation system equipped with a vacuum line or in a semi-batch reactor. When the reaction rate is quite high, the latter type reactor is convenient, but the former type is the standard reactor for water splitting in most cases. In figure 4, schematic of our conventional photocatalytic reactor is depicted. In a reaction using a closed gas-circulation system, photocatalyst powder is dispersed in a reaction solution by magnetic stirring, and the reaction system is evaporated to remove dissolved air in the reaction solution. After the evacuation, the vapor pressure of the reaction solution becomes equal to the pressure of gas phase, namely in the boiling point. To avoid boiling of the solution during photoirradiation, an inert gas, Ar typically used for the carrier of gas chromatograph, is introduced in the reaction system. However, even with an introduction of inert gas, photocatalytic reaction rates tend to decrease [12]. This is because an increased gas phase pressure slows bubble formation and desorption of product gases from the photocatalyst surface, which increases a chance to undergo a reverse reaction to regenerate water. If a photocatalyst has activity for the reverse reaction, introduction of inert gas in the reaction system results in the suppression of photocatalytic activity. To reduce or prevent this effect, the amount of inert gas introduction should be minimized, and a reactor should be well-cooled using a cooling-water flow.

For light sources, a high-pressure mercury lamp or a Xe lamp is usually employed. The former lamp is mainly used for UV light illumination, and the cut-off wavelength is 190, 300 and 400 nm when using all-quartz optics, Pyrex glass or NaNO_2_ aqueous solution filter, respectively. On the other hand, the Xe lamp is mostly employed for visible light irradiation. The wavelengths of light can be controlled by inserting filters.

For an analysis of produced gases in photocatalytic water splitting, generally, N_2_ should be detected in addition to H_2_ and O_2_ by gas chromatograph. In the use of nitride or oxynitride photocatalysts, N_2_ evolution can be an indication for the self-oxidation of photocatalyst, therefore, the detection of N_2_ is indispensable. In other case, the detection of N_2_ indicates a leakage of air from outside or an insufficient removal of dissolved air in the reaction solution. In a typical setting for gas chromatograph, an online gas-sampling system, thermal conductivity detector, molecular sieve 5A column and Ar carrier gas are employed.

## 4. Oxide photocatalyst for UV-light-induced water splitting

In the early stages of research on photocatalysis, TiO_2_ and other titanates were examined based on the idea that these materials have suitable band positions. However, the water splitting reaction did not proceed as successfully as was expected on the basis of the band positions. The main reason for these failures was the concurrence of the reverse reaction. However, this problem was solved mainly by improved cocatalyst structures and reaction conditions [8–13], and stoichiometric water splitting into H_2_ and O_2_ became possible. Then, various other materials were examined as photocatalysts in order to obtain much higher activity and develop useful knowledge.

Various oxide photocatalysts for overall water splitting have been developed since the 1980s. In some cases, the water splitting reaction proceeds with a relatively high quantum efficiency (QE) [14–16]. Typically, NaTaO_3_ doped with La offers the highest QE of 57% at 270 nm [15]. However, almost all of the developed oxide photocatalysts are wide-gap materials requiring UV light excitation. However, the solar spectrum mostly covers the visible and infrared regions, and therefore efficient solar energy utilization is not possible using these wide-gap oxides. After numerous studies of such oxide photocatalysts, the methodology and knowledge of how to perform overall water splitting became well-established, and subsequent research has aimed at developing visible-light sensitive materials for water splitting since approximately the year 2000.

By examining the available studies of various oxide photocatalysts, it can be found that the components usable as the main cationic species of a water splitting photocatalyst have one of two electronic states, i.e., a d^0^- or d^10^- electronic configuration. The former corresponds to the transition metal cations Ti^4+^, Zr^4+^, Nb^5+^, Ta^5+^, and W^6+^ [8–15, 17–21], while the latter corresponds to the typical metal cations Ga^3+^, In^3+^, Ge^4+^, Sn^4+^, and Sb^5+^ [16, 22–27]. In oxides based on these metal cations, the lower part of the conduction band mainly consists of the outermost d or s–p hybridized orbitals, while the upper part of the valence band consists of O2p orbitals in all cases. The energy levels of the VBM are located at approximately 3 eV (versus normal hydrogen electrode), and are similar for most of these oxides because they consist of the same constituent orbitals [28]. The bandgap energies for such oxides inevitably exceed 3 eV because of the excessively positive VBM levels if the CBM levels are more negative than the H^+^/H_2_ level. In other words, if the bandgap of the oxide becomes narrow enough to be capable of visible-light absorption, H_2_ evolution becomes impossible due to the more positive CBM level than H^+^/H_2_ level. A typical example of this case is WO_3_ (2.6 eV), which is well-employed as an efficient water oxidation photocatalyst or photoanode [29, 30]. Eventually, no visible-light sensitive photocatalyst for direct water splitting could be found among such d^0^- or d^10^-based oxides, and other strategies had to be considered for developing visible-light-sensitive photocatalysts.

## 5. Strategy for the development of photocatalysts for visible-light utilization

**Figure 5.**
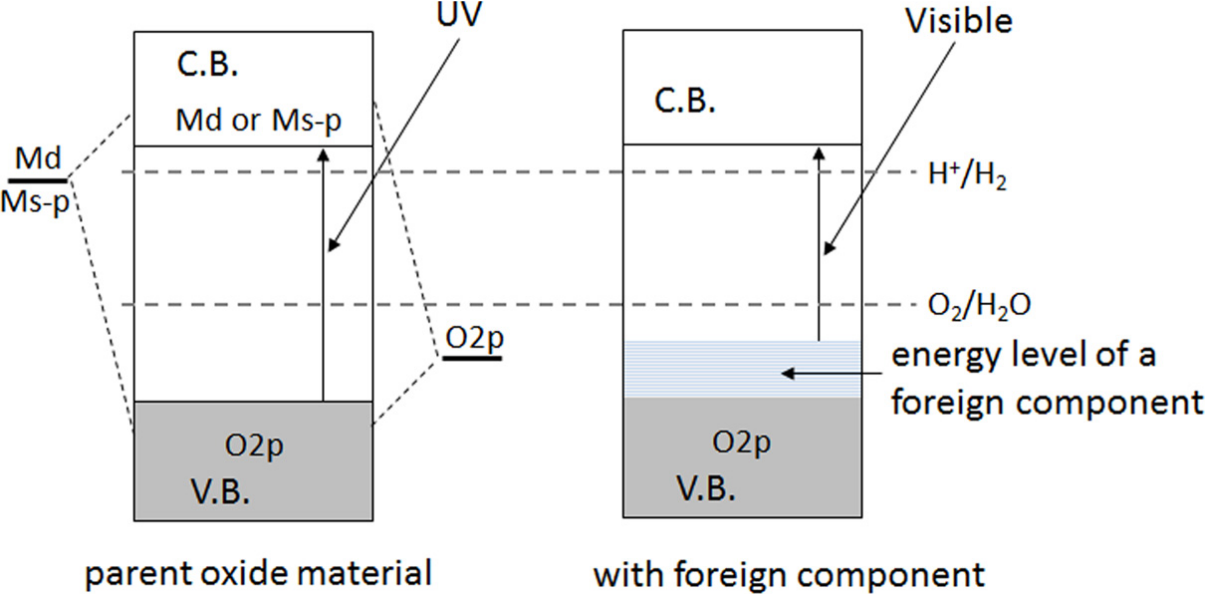
Strategy for designing visible-light-sensitive photocatalysts for water splitting.

As mentioned in the former section, d^0^-transition metal cations or d^10^-typical metal cations are the primary usable metal components for a water splitting photocatalyst because their orbitals can form a suitable conduction band. The aforementioned oxides have surplus energy in the side of water oxidation due to their excessively positive VBM levels. Therefore, most bandgap narrowing efforts have been directed toward the negative shift of the VBM level compared to the d^0^- and d^10^-based oxide photocatalysts. A reasonable strategy for accomplishing such a shift is the introduction of other constituent orbitals above the O2p state. For example, filled d-orbitals of a transition metal cation or filled s-orbitals of a typical metal cation can form an energy level above the O2p state, as schematically illustrated in figure 5. The introduction of foreign metal cations to wide-gap oxides is one such approach. Kudo *et al* have extensively investigated this approach, and have developed many successful examples [5, 31–34].

Another approach is to replace the oxygen in a metal oxide photocatalyst with different anionic components in order to introduce a new filled energy state at a more negative electrode potential than the O2p state, which is expected to shift the VBM toward the negative electrode potential. Certain chalcogenides and pnictides such as Cd (S, Se, Te) and Ga (P, As) are examples of such bandgap control. However, such non-oxides are subjected to photodissociation [35, 36], and have therefore not been usable as water splitting photocatalysts even though they do accomplish the desired bandgap narrowing. In contrast to these unstable non-oxide photocatalysts, we discovered that some metal nitride/oxynitride photocatalysts could avoid photodissociation and had a bandgap that enabled water splitting via visible-light excitation. In (oxy)nitride materials, N2p orbitals form a filled energy state at a more negative electrode potential than the O2p state, so that bandgap narrowing and visible-light absorption are possible. In this case, it is expected that the conduction band levels are not significantly affected because the cationic components are not changed [37]. Since N^3−^ has a similar ionic radius to that of O^2−^, partial or full substitution of N^3−^ for all or part of the O^2−^ in the oxide is possible in some cases.

We began researching oxynitride photocatalysts after the report of La_1−*x*_Ca_*x*_TaO_1+*x*_N_2−*x*_ as new pigments by Jansen and Letschert in 2000 [38]. Various transition metal (group IIIA–VIA) or typical metal (group IIB–VB) oxynitrides/nitrides are known. Among them, we have mainly examined oxynitrides based on Ti, Nb, Ta, Ga, and Ge as photocatalysts because many of these materials can maintain suitable valence states, i.e., d^0^- or d^10^-electronic configurations. The progress with regard to photocatalysts for overall water splitting based on metal oxynitrides is described in the following sections.

## 6. d^10^-type typical metal oxynitrides

Various typical metal oxides with d^10^-electronic configurations have been demonstrated to be usable as photocatalysts for efficient overall water splitting [40]. Thus, d^10^-typical metal based nitrides and oxynitrides were also expected to be usable for water splitting. Using these materials, successful progress toward efficient photocatalytic overall water splitting has been made.

### 6.1. Ge_3_N_4_ photocatalyst for UV-light-induced overall water splitting

In the study of d^10^-nitrides, a Ge_3_N_4_ photocatalyst was the first found to be capable of overall water splitting [39]. Ge_3_N_4_ is known to be polymorphic, exhibiting *α*, *β*, *γ*, and *δ* phases with different crystal structures [40–43]. Among these, the *β*-phase was demonstrated to be active for overall water splitting when combined with a suitable cocatalyst.

**Figure 6.**
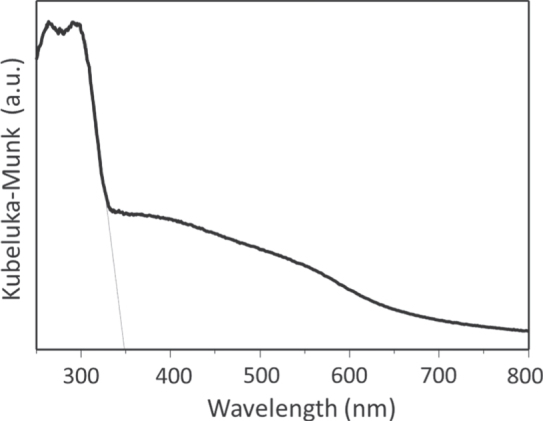
UV-visible diffuse reflectance spectrum of *β*-Ge_3_N_4_.

Typically, *β*-Ge_3_N_4_ can be synthesized by heating a GeO_2_ powder under a dry NH_3_ flow at 1123–1173 K for ∼20 h. A lower heating temperature or a shorter heating duration tends to generate a mixture of *α*- and *β*-phases, with a higher *α*-phase content. As shown in the UV-visible diffuse reflectance spectrum (figure 6), a single phase of *β*-Ge_3_N_4_ exhibits a broad absorption that can be divided to two components. The first is a strong absorption band in the UV region (>ca. 350 nm), and the other is a weak absorption tailing into the longer wavelength region. The former absorption is attributed to genuine bandgap excitation, while the latter is likely attributable to an excitation related to a defect level.

**Figure 7.**
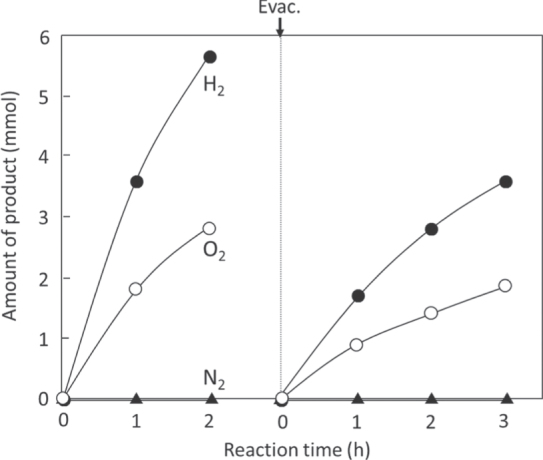
Time course of H_2_ and O_2_ evolution on RuO_2_-loaded *β*-Ge_3_N_4_ under UV light irradiation. Catalyst 0.5 g, aqueous H_2_SO_4_ solution (pH = 0) 390 ml, inner-irradiation-type quartz reaction cell, high-pressure mercury lamp (450 W).

When RuO_2_-cocatalyst nanoparticles (20–50 nm in size) are dispersed on the surface of particulate *β*-Ge_3_N_4_ (0.5–2 *μ*m in size), simultaneous H_2_ and O_2_ evolution occur under UV light irradiation. H_2_ and O_2_ evolve in an almost stoichiometric ratio for water splitting, as shown in figure 7. This is the first example of overall water splitting using a non-oxide photocatalyst, which demonstrates that an (oxy)nitride material can be utilized as a photocatalyst for water splitting. However, this photocatalyst is active only under UV-light excitation, and the absorption band in the visible-light region is not functional for water splitting. In the abovementioned strategy, it is expected that the introduction of nitrogen narrows the bandgap compared to that of the corresponding oxide. Although the bandgap of Ge_3_N_4_ is not narrow enough to absorb visible light, introducing nitrogen certainly narrows its bandgap, compared that of GeO_2_ (>5.2 eV) [44, 45]. The discovery of the Ge_3_N_4_ photocatalyst confirmed the validity of our strategy and encouraged us to continue our (oxy)nitride research.

Although overall water splitting was achieved on a d^10^-type nitride, Ge_3_N_4_, visible light utilization was not realized because of its larger bandgap, so the survey of different material types with suitable bandgap energies for the absorption of visible light continued. GaN is a well-known and well-used material for optoelectronic devices [46, 47]. It also has a wide bandgap (3.4 eV), although the bandgap can be made narrower than that of Ga_2_O_3_ (4.7 eV) [48] by introducing nitrogen. GaN was also demonstrated to be active for water splitting via bandgap excitation after proper modifications [49, 50]. The nitrides of other typical metals, e.g., indium and tin, tend to be unsuitable. Indium nitride can maintain a d^10^-electronic configuration, but the bandgap is narrower than the theoretical limit of 1.23 eV [51], whereas tin nitride cannot maintain a d^10^-electronic configuration due to their partial reduction by ammonia during their synthesis [52]. In the end, no suitable materials for visible-light water splitting were found in the group of d^10^-type binary nitrides. Therefore, complex (oxy)nitrides consisting of more than one metal were subsequently examined.

### 6.2. Visible-light-sensitive GaN:ZnO solid solution as a photocatalyst for overall water splitting

A complex oxynitride consisting of Ga, Zn, O, and N was examined as a new photocatalyst in an attempt to devise a band structure suitable for absorbing visible light. The Zn component was introduced to modify the band structure according to the following idea. For II–VI semiconductors, it is predicted that interactions between the valence band constituents, e.g., repulsion between O2p and Zn3d electrons, shift the VBM toward the negative electrode potential [53]. It is expected that N2p–Zn3d repulsion could cause a negative shift in the VBM, resulting in bandgap narrowing. A crystalline (oxy)nitride compound based on Ga and Zn was not yet known, so the synthesis of a new compound was attempted, beginning with the examination of a series of solid solutions between GaN and ZnO. GaN [54] and ZnO [55] have the same wurtzite crystal structure, and their lattice parameters are quite close, which suggests that they are suitable for the formation of a solid solution.

**Figure 8.**
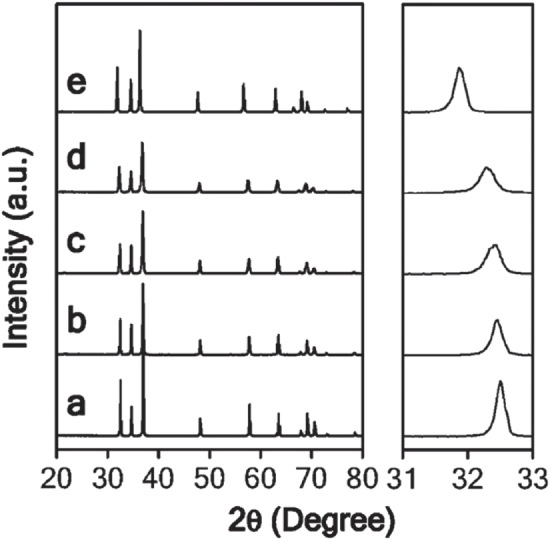
XRD patterns for GaN:ZnO solid solutions with various compositions. (a) GaN (ref), (b) GaN:ZnO (Zn 3.4 at%), (c) GaN:ZnO (Zn 6.4 at%), (d) GaN:ZnO (Zn 13.3 at%), and (e) ZnO. (Reproduced with permission from [56]. Copyright 2005 American Chemical Society).

Typically, a solid solution can be synthesized by nitriding a mixture of Ga_2_O_3_ and ZnO (Ga: Zn = 1: 1 molar ratio) under a dry ammonia flow at 1223 K for various durations [56]. The products exhibit x-ray diffraction (XRD) patterns assignable to the wurtzite structure and similar to those of GaN and ZnO (figure 8). The diffraction peak positions for the as-synthesized samples were located between those of GaN and ZnO, and were shifted to the GaN-side with increasing nitridation duration. Elemental analysis carried out using inductively coupled plasma optical emission spectroscopy (ICP-AOS) and the combustion method revealed that the Zn content of the samples decreased with increasing nitridation duration. Moreover, the ratios of Ga to N and Zn to O were both close to 1. These results indicate that the products can be regarded as solid solutions of GaN and ZnO, and can be generally formulated as (Ga_1−*x*_Zn_*x*_)(N_1−*x*_O_*x*_), although the actual compositions of the products deviated slightly from their intended stoichiometry. In this manuscript, the solid solution is denoted as GaN:ZnO for simplicity. The results of the ICP-AOS analysis indicated that the ratio of Ga to Zn is variable, and depends on the nitridation duration. Under the examined nitridation conditions, ZnO can be reduced to Zn, which can then be lost by volatilization. In practice, a substantial portion of the ZnO in the feedstock was not incorporated into the products under the examined synthetic conditions. Therefore, the composition of the products can be controlled by adjusting the nitridation duration. The crystal structure of the GaN:ZnO solid solution mentioned above was confirmed in detail by Rietveld refinement [57, 58].

**Figure 9.**
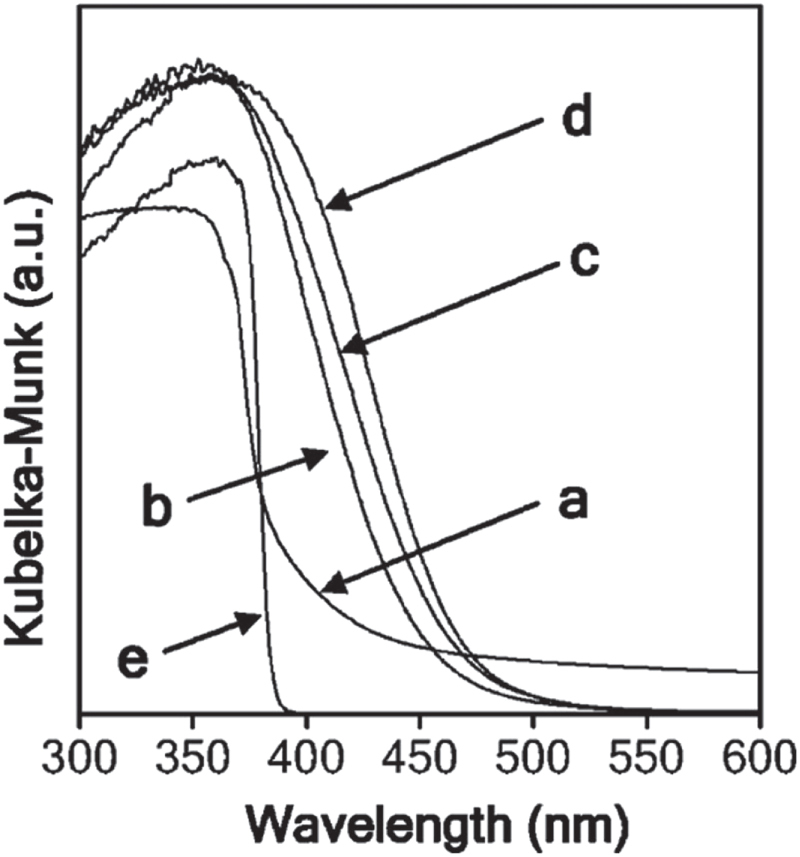
UV-visible diffuse reflectance spectra of GaN:ZnO solid solutions with various compositions. (a) GaN (ref), (b) GaN:ZnO (Zn 3.4 at%), (c) GaN:ZnO (Zn 6.4 at%), (d) GaN:ZnO (Zn 13.3 at%), and (e) ZnO. (Reproduced with permission from [56]. Copyright 2005 American Chemical Society).

As can be seen from the UV-visible diffuse reflectance spectra (figure 9), the primary absorption bands lie in the UV region for both GaN and ZnO, whereas the absorption band edges for the solid solutions extend into the visible region. The absorption edges for the solid solutions shift towards red with increasing Zn and O content, as far as examined in the side of Ga and N-rich compositions. The obtained solid solutions were yellow, while GaN and ZnO are light gray and white, respectively. This result is certainly indicative of a change in electronic band structure. The interpretation of the change in optical absorption was investigated by DFT calculations, which gave an explanation that was contradictory to the hypothesis described above [59].

**Figure 10.**
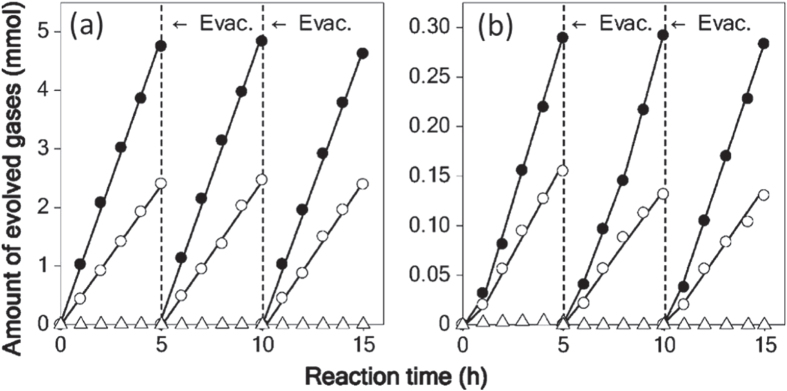
Time courses of H_2_ and O_2_ evolution on RuO_2_-loaded GaN:ZnO under (a) UV light irradiation and (b) visible light irradiation. Catalyst 0.3 g, aqueous H_2_SO_4_ solution (pH = 3) 390 ml, inner-irradiation-type Pyrex reaction cell, with and without an aqueous NaNO_2_ solution filter, high-pressure mercury lamp (450 W). (●) H_2_, (○) O_2_, (△) N_2_. (Reproduced with permission from [56]. Copyright 2005 American Chemical Society).

The as-obtained GaN:ZnO solid solutions were examined for photocatalytic water splitting capability [56, 60]. RuO_2_ nanoparticles as a cocatalyst were dispersed on the surface of the solid solutions similarly to the case of the Ge_3_N_4_ photocatalyst. The photocatalytic activities for water splitting on GaN:ZnO solid solutions for various compositions and with various loading amounts of RuO_2_ cocatalyst were examined in detail. Among these, (Ga_1−*x*_Zn_*x*_)(N_1−*x*_O_*x*_) with *x* = 0.12 loaded with a 5 wt% RuO_2_ showed the highest activity. H_2_ and O_2_ evolved steadily and stoichiometrically upon light irradiation, and the reaction continued even under visible light irradiation, as shown in figure 10. The optical absorption edge and bandgap for the solid solution with this composition were 482 nm and 2.68 eV, respectively. This was the first reproducible example of visible-light induced overall water splitting on a single photocatalyst system.

Although visible-light driven overall water splitting was demonstrated using GaN:ZnO solid solutions, the photocatalytic activity was low. The QE at this stage (*x* = 0.12, RuO_2_ 5 wt% loading, pH = 3) was 0.23% at 410 nm. Improvement of the cocatalyst was attempted to enhance the photocatalytic activity of this photocatalyst. A range of transition metal oxides was examined as cocatalysts for the GaN:ZnO photocatalyst [61]. The cocatalysts were loaded by impregnation from aqueous solutions containing the corresponding metal salts followed by heating in air. Some metal oxides, e.g., Ni, Ru, Rh, Pt, and Ir somewhat promoted overall water splitting, as summarized in the left column of table 1. The loading of Ru-oxide cocatalyst resulted in the highest activity of the binary oxides examined.

**Table 1.** Photocatalytic activities of GaN:ZnO loaded with various cocatalysts for water splitting under UV light irradiation.

				Cr-coloading		
Metal oxides		Activity/mmol h^*−*1^		Cr-loading amount/wt%	Activity/mmol h^*−*1^	
Metal	Loading amount/wt%	H_2_	O_2_		H_2_	O_2_
None	—	0	0	—	—	—
Cr	1	0	0	—	—	—
Fe	1	0	0	1	73	36
Co	1	2.0	0	1	48	24
Ni	1.25	126	57	0.125	685	336
Cu	1	2.0	0	1	585	292
Ru	1	71	27	0.1	181	84
Rh	1	50	1.6	1.5	3835	1988
Pd	1	1.0	0	0.1	205	96
Ag	1	0	0	1	11	2.3
Ir	1.5	9.3	3.1	0.1	41	17
Pt	1	0.9	0.4	1	775	357

Catalyst 0.3 g, distilled water, high pressure mercury lamp (450 W), inner irradiation type reaction cell made of Pyrex [61].

Ternary oxide cocatalysts were also examined, as summarized in the right column of table 1. The water splitting activities of GaN:ZnO photocatalyst can be remarkably enhanced by co-loading with Cr oxide. Loading Cr oxide alone is not at all effective for the promotion of water splitting. However, the addition of Cr oxide as a second cocatalyst component did promote overall water splitting, and a similar effect was observed for various pairs of metal oxide cocatalysts. With binary oxide loading, the amount of evolved O_2_ tended to be deficient from the stoichiometric amount for water splitting, but in many cases, the ratio of evolved H_2_ and O_2_ became closer to stoichiometric after co-loading with Cr-oxide. The Rh and Cr oxide co-loaded sample had the highest activity of all ternary oxides examined. H_2_ and O_2_ evolution proceeded in a stoichiometric ratio at a constant reaction rate on the Rh and Cr oxide co-loaded sample. Detailed characterization of the Rh + Cr oxide cocatalyst by x-ray photoelectron spectroscopy, x-ray absorption fine structure, and transmission electron microscopy revealed that a mixed oxide structure was formed after loading by the impregnation method [62]. The Rh and Cr species in the cocatalyst both have a +3 valence state after loading and exist as a complex oxide of Rh_2_O_3_ and Cr_2_O_3_, generally formulated as Rh_2−*y*_Cr_*y*_O_3_. Both of these oxides tend to have the same corundum structure, which could be responsible for the formation of the mixed oxide structure. The development of the Rh_2−*y*_Cr_*y*_O_3_ cocatalyst resulted in a remarkable enhancement in the photocatalytic activity of GaN:ZnO (QE = 5.2% at 410 nm) compared with the case of Ru oxide cocatalyst loading.

### 6.3. Role of Cr-species in the Rh–Cr bimetallic cocatalyst for promoting overall water splitting

The discovery of the ternary oxide cocatalyst resulted in a remarkable enhancement of photocatalytic water splitting. Notably, the addition of Cr oxide as a second cocatalyst component greatly improved overall water splitting performance. This effect is widely applicable to various combinations with other cocatalyst components, which indicates that Cr oxide plays an essential role in overall water splitting. Therefore, understanding this phenomenon should provide important knowledge and enable further progress. The role of Cr oxide addition is next introduced in relation to an extended form of the Rh_2−*y*_Cr_*y*_O_3_ cocatalyst.

Cr oxide loading alone does not promote water splitting, as mentioned above. Therefore, it is deduced that the role of Cr oxide is to assist the function of the paired cocatalyst components. Generally, the cocatalyst components, e.g., Ni, Ru, Rh, and Pt, are known to promote H_2_ evolution due to their low overpotentials. However, they are also active for the oxygen reduction reaction (ORR), which corresponds to the backward reaction of water splitting. From the viewpoint of thermodynamics, the ORR is predominant over the water splitting reaction, and the water splitting reaction does not proceed efficiently when these reactions compete with each other. The main role of Cr oxide addition is to prevent the ORR without suppressing H_2_ evolution [63], and this was demonstrated by the following experiments.

First, a Rh cocatalyst was loaded on a GaN:ZnO photocatalyst by photodeposition. At that point, photocatalytic overall water splitting did not proceed efficiently due to the rapid ORR on the surface of the Rh cocatalyst. Then, Cr_2_O_3_ was loaded onto the Rh/GaN:ZnO by photodeposition from an aqueous solution containing K_2_CrO_4_ as precursor. There, photoexcited electrons from GaN:ZnO reduce 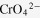 ions on the surface of the Rh, while positive holes oxidize H_2_O on the bare GaN:ZnO surface. Using this two-step photodeposition, Cr_2_O_3_ can be deposited selectively on the surface of the cocatalyst, creating a core/shell-structured cocatalyst [64]. The thickness of the Cr_2_O_3_ shell is usually about 2 nm. After loading the Rh/Cr_2_O_3_-core/shell cocatalyst, photocatalytic overall water splitting proceeds efficiently, which is similar to the case of Rh_2−*y*_Cr_*y*_O_3_ loading. This effect of Cr_2_O_3_ photodeposition is also applicable to certain other metal cores.

**Figure 11.**
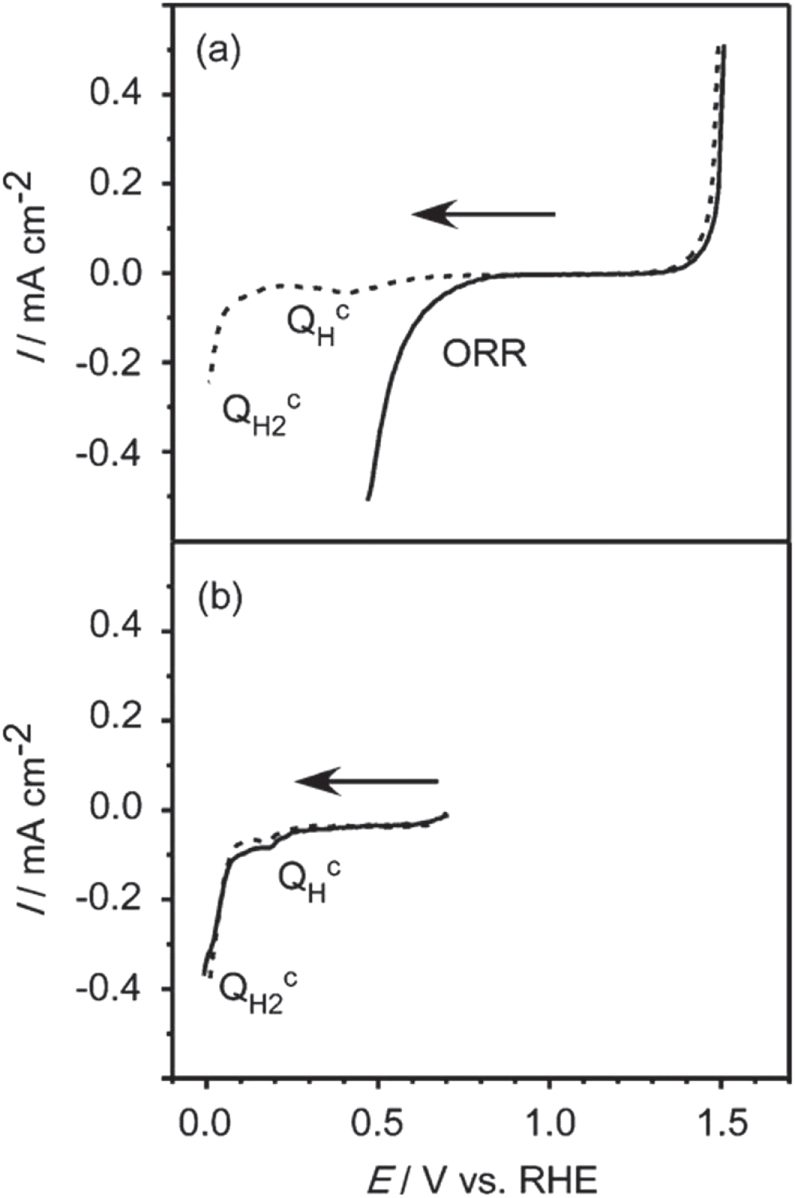
Linear-sweep voltammograms for (a) bare and (b) Cr_2_O_3_-coated Rh electrodes in 0.5 M Na_2_SO_4_ aqueous solution adjusted to pH 3.6 with H_2_SO_4_ under Ar (dashed line) and O_2_ bubbling (solid line) (scan rate, 5 mV s^−1^). (Reproduced with permission from [65]. Copyright 2009 American Chemical Society).

**Figure 12.**
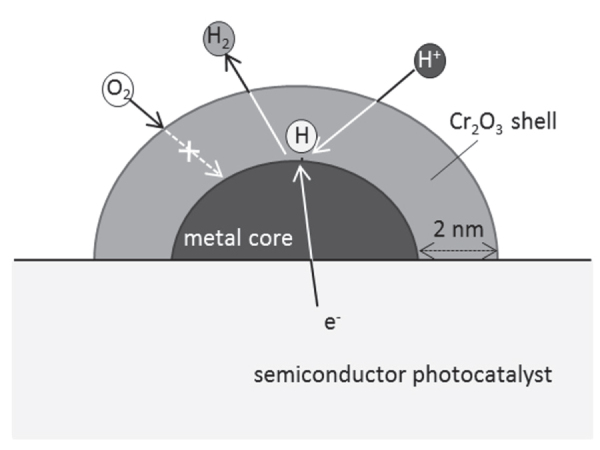
Schematic model of the function of metal-core/Cr_2_O_3_-shell cocatalyst for the promotion of overall water splitting.

The two different types of structures have been known for Rh–Cr bimetallic cocatalyst system as mentioned above. One is the complex oxide of Rh_2_O_3_ and Cr_2_O_3_ loaded by co-impregnation, while the other is the Rh-core/Cr_2_O_3_-shell structure loaded by two-step photodeposition. Their structures are not identical, nevertheless, both of them show the similar function. Thus, it is considered that the key structures for their functionalities are essentially identical. Then the role of Cr-addition was studied using the latter type because of its well-defined structure. Here, an electrochemical study using a model structured electrode was performed [65]. The entire surface of a Rh metal electrode was coated with a thin Cr_2_O_3_ layer by electroplating from an aqueous K_2_CrO_4_ solution, which imitated the core/shell-cocatalyst structure. The redox properties of this model electrode were examined in comparison with those of a bare metal electrode. Figure 11 shows linear-sweep voltammograms of Rh and Cr_2_O_3_-coated Rh electrodes under Ar or O_2_ bubbling. In the absence of O_2_, the bare Rh and Cr_2_O_3_-coated Rh electrodes both generated a cathodic current attributable to H_2_ evolution at approximately 0 V. In the presence of O_2_, the bare Rh electrode generated a cathodic current attributable to the ORR from 0.85 V, whereas the ORR was suppressed by the Cr_2_O_3_ coating. Furthermore, the Cr_2_O_3_-coated electrode generated a similar cathodic current attributed to H_2_ evolution in the presence and absence of O_2_, indicating that the Cr species does not significantly affect H_2_ evolution. These results indicate that the Cr_2_O_3_-coated electrode is inert to the ORR but active for H_2_ evolution, and that H_2_ evolution occurs not on the Cr_2_O_3_ layer but on the Rh surface. Cr_2_O_3_ layer is an insulator for electron-transport, thereby, this layer thickness does not increase above 2 nm. The role and function of the Cr species for enhancing water splitting are schematically shown in figure 12. On the Cr_2_O_3_-coated electrode, the Rh/Cr_2_O_3_ interface provides active sites for H_2_ evolution. The deposited Cr_2_O_3_ thin layer is hydrated in water, and the supply of H^+^(H_2_O) to the Cr_2_O_3_/Rh interface is possible, whereas the diffusion of O_2_ to the Cr_2_O_3_/Rh interface is prevented by the Cr_2_O_3_ layer. The Cr_2_O_3_ layer, in summary, functions like a molecular sieve, and the selective permeability of this layer enables the selective prevention of the ORR, which results in efficient overall water splitting. This work demonstrates the importance of the cocatalyst in the control of surface redox reactions for achieving efficient overall water splitting. Moreover, the function of this special surface reaction control was further extended, leading to successful overall water splitting on a transition metal oxynitride photocatalyst. The detail of this progress is given in the next section.

## 7. d^0^-transition metal oxynitride photocatalysts

### 7.1. Various d^0^-transition metal oxynitrides as visible-light-sensitive photocatalysts

Visible-light-induced overall water splitting was realized with the discovery of the GaN:ZnO photocatalyst, and this photocatalyst system has well been developed. However, the longest wavelength usable by this photocatalyst system is limited to around 500 nm because the absorption band edges of GaN:ZnO solutions are at approximately 450–500 nm. In this case, only a small portion of the solar spectrum can be utilized, and further extension of the usable wavelength region is needed in order to efficiently harness solar energy. To achieve this aim, d^0^-transition metal oxynitrides are suitable candidates because many of them have absorption band edges above 600 nm. However, no one has yet succeeded in direct overall water splitting on a transition metal oxynitride with an absorption edge at up to 600 nm.

**Figure 13.**
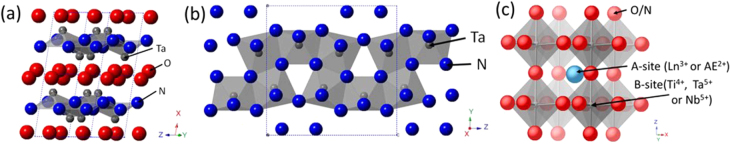
Crystal structures of TaON (a), Ta_3_N_5_ (b), and perovskite oxynitride (c).

Various transition metal oxynitrides based on Ti^4+^, Nb^5+^, and Ta^5+^ have been intensively examined as photocatalysts for water splitting over the past 15 years [6, 66]. Figure 13 shows the crystal structures of representative transition metal oxynitrides that we have examined so far. TaON has a bandgap energy of 2.4–2.5 eV, absorbs light with wavelengths shorter than ca. 500 nm, has a baddeleyite structure, and is isostructural with monoclinic ZrO_2_. [67] Ta_3_N_5_ is a nitride consisting of corner- or edge-sharing TaN_6_ octahedra, has an anosovite structure with a bandgap energy of 2.1 eV, and is capable of optical absorption up to 600 nm [68]. Except for these two materials, most of the oxynitrides examined have a perovskite structure, which is generally formulated as ABX_3_. The A-site is occupied by relatively large cations, AE^2+^ (Ca^3+^, Sr^2+^, Ba^2+^) and Ln^3+^ (La^3+,^ Nd^3+^), while the B-site is occupied by smaller cations such as Ti^4+^, Nb^5+^, and Ta^5+^. The X-site is occupied by anionic components, O^2−^ and N^3−^. The O/N ratio in a perovskite compound is determined by the valences of the involved cations, so that the sum of the O^2−^ and N^3−^ charges is balanced by that of the cations. For example, the O:N ratio in AETaO_2_N compounds is 2:1, with a total valence of −7, whereas AE and Ta formally have valences of +2 and +5, respectively. When the AE^2+^ in these compounds is fully substituted by Ln^3+^, the O:N ratio in the product changes to 1:2 to maintain the charge balance, as represented by LnTaON_2_. Thus the O:N ratio in the product is controllable.

**Figure 14.**
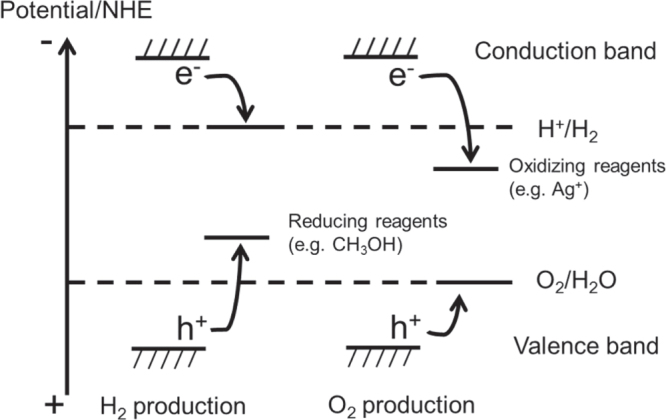
Basic principles of photocatalytic reactions in the presence of sacrificial reagents.

These d^0^-transition metal oxynitrides have been examined as visible light responsive photocatalysts. These compounds have bandgap energies of 1.8–2.5 eV and absorb light with wavelength shorter than ca. 500–700 nm. It was confirmed that these compounds have some photocatalytic capability: overall water splitting has not been possible on these compounds, even with modification by already-known cocatalysts, but H_2_ and/or O_2_ evolution is possible in the presence of a proper sacrificial reagent under visible light irradiation [5, 6]. Sacrificial H_2_ and O_2_ evolutions are employed as test reactions to judge whether or not a photocatalyst has the potential for overall water splitting. The basic concept for the use of sacrificial reactions is schematically depicted in figure 14. When the photocatalytic reaction is conducted in the presence of an electron donor such as methanol, photogenerated holes in the valence band predominantly oxidize methanol rather than H_2_O, thus facilitating H^+^ reduction by conduction band electrons if the CBM of the photocatalyst is located at a more negative electrode potential than the H^+^ reduction potential. On the other hand, in the presence of an electron acceptor such as silver cations, photogenerated electrons in the conduction band predominantly reduce Ag^+^ rather than H^+^, thereby accelerating water oxidation by valence band holes if the VBM of the photocatalyst is located at a more positive electrode potential than the water oxidation potential. If both H_2_ and O_2_ evolution are possible in the presence of such sacrificial reagents, this proves that the photocatalyst has the potential for overall water splitting.

The photocatalytic activities of several representative d^0^-transition metal oxynitrides in such sacrificial conditions are summarized in table 2, along with their bandgap energies [69–73]. Among these, TaON, Ta_3_N_5_, and LaTiO_2_N certainly have some photocatalytic activity for H_2_ and O_2_ evolution. On the other hand, AETaO_2_N and LnTaON_2_ can only evolve H_2_. The photocatalytic activities shown in the table are results obtained in the early stages of our research. We subsequently found that the H_2_ and O_2_ evolution activities tend to change depending on the synthetic conditions, which suggests that a minor difference in the composition of a product can affect its photocatalytic activity. At the present stage, insufficient experimental data explaining such phenomena have been obtained.

**Table 2.** Photocatalytic activities of (oxy)nitrides with d^0^ electronic configuration for H_2_ or O_2_ evolution in the presence of sacrificial reagents under visible light (*λ* ≥ 420 nm)^a^.

		Activity/*μ*mol h^−1^		
Photocatalyst	Band gap^b^/eV	H_2_^c^	O_2_^d^	Reference
CaNbO_2_N	1.9	1.5	46	[70]
LaTiO_2_N	2.0	30	41	[71]
Ca_0.25_La_0.75_TiO_2.25_N_0.75_	2.0	5.5	60	[71]
TaON	2.5	20	660	[68]
Ta_3_N_5_	2.1	10	420	[69]
CaTaO_2_N	2.4	15	0	[72]
SrTaO_2_N	2.1	20	0	[72]
BaTaO_2_N	1.9	15	0	[72]
LaTaON_2_	2.0	20	0	[70]

a Reaction conditions: 0.2–0.4 g of catalyst, 200 ml of aqueous solution containing sacrificial reagents, 300 W xenon lamp light source, top irradiation-type reaction vessel made of Pyrex, 420 nm cutoff filter.

b Estimated from onset wavelength of diffuse reflectance spectra.

c Loaded with Pt as a cocatalyst; reacted in aqueous methanol solution (10 vol%).

d Reacted in aqueous silver nitrate solution (0.01 M) [6].

If either of the sacrificial H_2_ or O_2_ evolution capabilities is deficient in a photocatalyst, direct water splitting is impossible. However, even for such a photocatalyst, overall water splitting is possible by constructing a so-called Z-scheme photocatalytic system with two-step excitation. In a system of this kind, H_2_ is evolved on one type of semiconductor photocatalyst, while O_2_ is evolved on another. In this case, positive holes and excited electrons remain in the valence band of one type of semiconductor photocatalyst and the conduction band of another, respectively. By creating a short-circuit between these remaining charges, overall water splitting can proceed. The details of Z-scheme photocatalytic water splitting have been reported in previous reviews [5, 74]. Therefore, only direct overall water splitting is discussed here.

Although it has been confirmed that some d^0^-transition metal oxynitrides can produce H_2_ [71–73] and O_2_ [69–72] in the presence of a sacrificial electron donor and acceptor, respectively, overall water splitting has not been possible. The two sacrificial reactions are a prerequisite for overall water splitting, but do not necessarily lead to successful overall water splitting. This is because the requirements for sacrificial reactions and overall water splitting are not identical. Bandgap narrowing makes overall water splitting more difficult because the driving force for the targeted reaction becomes smaller. Therefore, more advanced design and structural control of the photocatalyst are needed to perform overall water splitting.

One possible approach toward the design of active photocatalysts for overall water splitting is the substitution of certain foreign components for the parent components. This could change electronic parameters such as the bandgap energy and position, which should impact photocatalysis. Various substitutions are known to be possible for the abovementioned transition metal oxynitrides. For perovskite-type compounds, the A-site can be replaced with AE^2+^(Ca^2+^(134 pm), Sr^2+^(144 pm), Ba^2+^(161 pm)), Ln^3+^(La^3+^(136 pm), Nd^3+^(127 pm)), or alkaline metal cations (Na^+^(139 pm), K^+^(164 pm)). [37, 74, 75] On the other hand, the B-site of the perovskite can be replaced by Mg^2+^(72 pm), Sc^3+^(74.5 pm), Zr^4+^(72 pm), or Hf^4+^(71 pm). Mg^2+^ and Sc^3+^ occupy the B-site, although other AE^2+^ and Ln^3+^ occupy the A-site because of the smaller ionic radii of Mg^2+^ and Sc^3+^ [75, 76]. For baddeleyite and anosovite compounds, the Ta^5+^-site can be substituted by cations of similar size, e.g. Mg^2+^, Sc^3+^, Zr^4+^, or Hf^4+^ [77–79]. This selectivity for site occupancy is mainly determined by the similarity in ionic radii between the parent and foreign metal cations.

Various combinations of foreign components and parent compounds have been examined, and some examples of improved photocatalysis have been reported [77, 80–84]. However, in all of these reports, the photocatalytic performance was evaluated only using sacrificial reagents. Although the photocatalytic activities obtained under sacrificial conditions were enhanced by compositional modifications, this would not necessarily lead to successful overall water splitting. This is because the requirements for sacrificial reactions and water splitting are not entirely identical, as mentioned above. Therefore, we believed that a different approach should also be employed to achieve overall water splitting. Very recently, we discovered that one of the combinations of parent oxynitride compounds and foreign components has the capability for simultaneous H_2_ and O_2_ evolution from pure water under visible light irradiation. Furthermore, we devised a novel surface modification method, which resulted in stable and steady overall water splitting. The development of this new transition metal oxynitride photocatalyst for visible-light-induced overall water splitting is described below.

### 7.2. A complex perovskite oxynitride, LaMg_*x*_Ta_1−*x*_O_1+3*x*_N_2−3*x*_ (*x* ≥ 1/3), as a new type of photocatalyst with a wider range of visible light utilization

**Figure 15.**
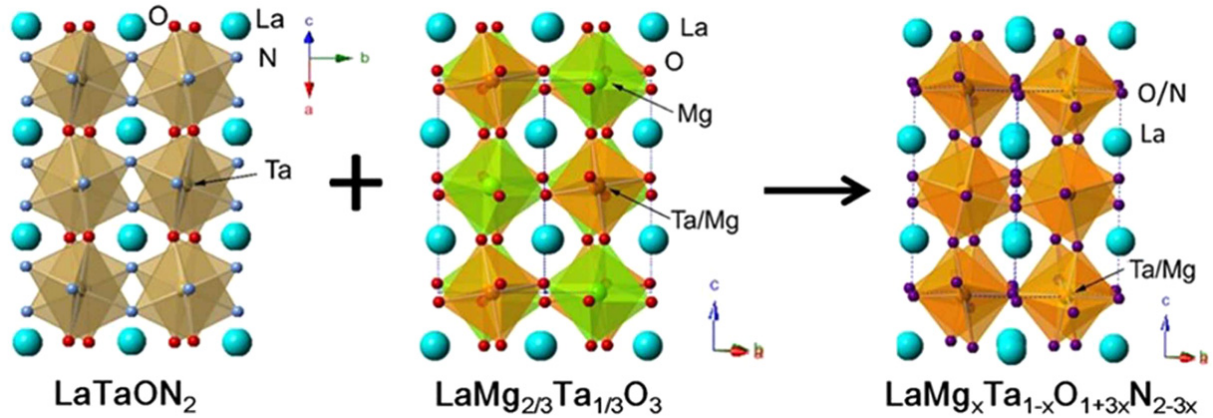
Crystal structures of LaTaON_2_, LaMg_2/3_Ta_1/3_O_3_, and LaMg_*x*_Ta_1−*x*_O_1+3*x*_N_2−3*x*_.

One of the perovskite oxynitrides introduced above, with the composition LaTaON_2_, was used as a base material to create LaMg_*x*_Ta_1−*x*_O_1+3*x*_N_2−3*x*_ compounds. LaTaON_2_ is unable to perform overall water splitting. Moreover, O_2_ evolution in the presence of a sacrificial electron acceptor is not possible [71]. However, compositional tuning of this material modifies its photocatalytic activity, leading to simultaneous H_2_ and O_2_ evolution under non-sacrificial conditions. LaTaON_2_ has a perovskite structure with La^3+^ and Ta^5+^ at the A- and B-sites, respectively [85]. LaMg_2/3_Ta_1/3_O_3_ is isostructural with LaTaON_2_, with two-thirds of the Ta-sites in LaTaON_2_ replaced by Mg^2+^ [86]. Compositional tuning was conducted by forming a series of solid solutions between LaTaON_2_ and LaMg_2/3_Ta_1/3_O_3_, with the general formula LaMg_*x*_Ta_1−*x*_O_1+3*x*_N_2−3*x*_. The crystal structures of the LaMg_*x*_Ta_1−*x*_O_1+3*x*_N_2−3*x*_ series are schematically depicted in figure 15. The synthesis of the solid solutions and their structural analysis have been reported, but they have not been examined as photocatalytic materials [87]. We recently discovered that a photocatalyst of this kind is active for overall water splitting under visible light with wavelengths of up to 600 nm [88].

**Figure 16.**
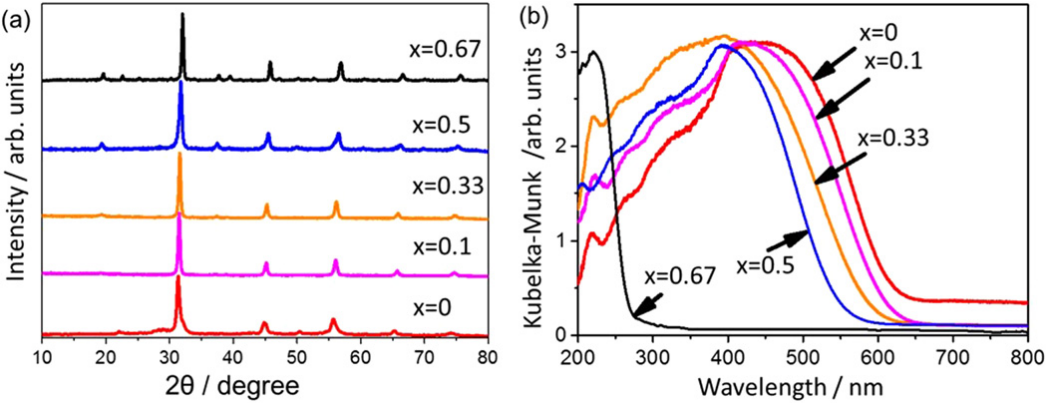
XRD patterns (a) and UV-visible diffuse reflectance spectra (b) for LaMg_*x*_Ta_1−*x*_O_1+3*x*_N_2−3*x*_.

LaMg_*x*_Ta_1−*x*_O_1+3*x*_N_2−3*x*_ can be synthesized by thermal ammonolysis of the corresponding oxides. The oxide precursors are prepared via a molecular route called the citric acid method [89]. This method produces an oxide precursor wherein each component can be intimately mixed, and this precursor is suitable for the production of multicomponent crystalline oxynitrides. Typically, the nitrided products can be obtained by heating at 1223 K for 7 h under dry ammonia flow (100 ml min^−1^). The nitrided products exhibit patterns similar to that for the perovskite structure, regardless of the different Mg/Ta ratios in the precursors (see figure 16(a)). The peak position shifts toward higher diffraction angles with increasing *x*. Thus, a series of LaMg_*x*_Ta_1−*x*_O_1+3*x*_N_2−3*x*_ solid solutions can be synthesized by changing the Mg/Ta ratio in the precursor.

**Figure 17.**
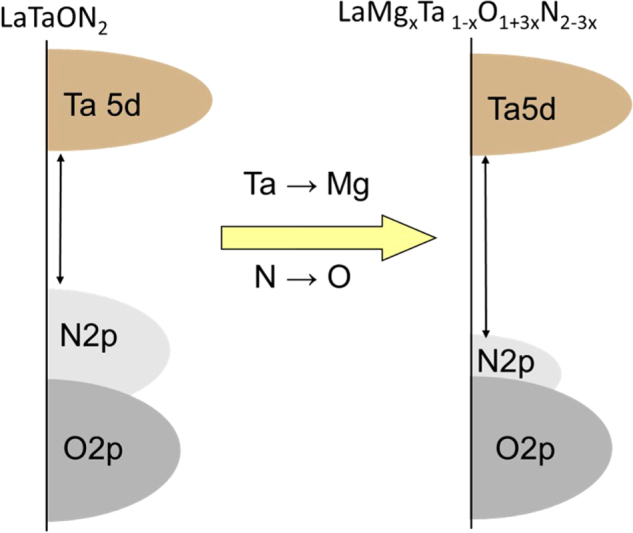
Schematic of band engineering by compositional tuning.

The wavelength of the absorption edge blue-shifts monotonically from 640 to 560 nm with increasing *x* from 0 to 0.5 (see figure 16(b)). Typically, the bandgap energies of LaMg_*x*_Ta_1−*x*_O_1+3*x*_N_2−3*x*_ with *x* = 0, 0.33, and 0.67 are 1.93, 2.08, and 4.59 eV, respectively, indicating bandgap widening with increasing *x*. The observed change in bandgap energy can be interpreted as follows. For these nitrided solid solutions, the lower part of the conduction band mainly consists of Ta5d orbitals, while the upper part of the valence band consists predominantly of N2p orbitals with a small contribution from O2p orbitals. The increase in Mg content decreases the N/O ratio, which shifts the VBM downward. The concept for the band engineering by compositional tuning is schematically illustrated in figure 17. Thermodynamically, this shift in the VBM level would be advantageous, at least for water oxidation. The increase in bandgap energy reduces the overlap of the absorption band with the solar spectrum, but enough of the visible light would still be usable for compositions with low Mg content.

**Figure 18.**
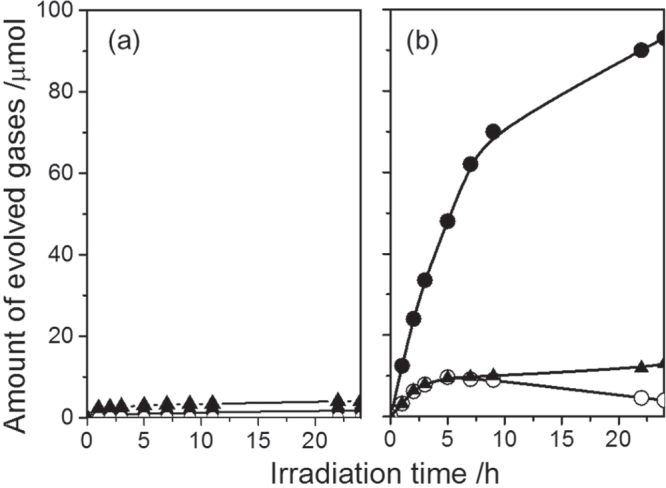
Gas evolution during water splitting on the Rh_*y*_Cr_2−*y*_O_3_/LaTaON_2_ (a) and on Rh_y_Cr_2−*y*_O_3_/LaMg_1/3_Ta_2/3_O_2_N (b). Catalyst 0.2 g, pure water 400 ml, inner-irradiation-type Pyrex reaction cell, high-pressure mercury lamp (450 W). (●) H_2_, (○) O_2_, (▲) N_2_.

Photocatalytic activities of LaMg_*x*_Ta_1−*x*_O_1+3*x*_N_2−3*x*_ for water splitting were examined. Figure 18 shows the typical time courses of H_2_ and O_2_ evolution from pure water under UV light (*λ* ≥ 300 nm) irradiation of Rh_2−*y*_Cr_*y*_O_3_/LaMg_*x*_Ta_1−*x*_O_1+3*x*_N_2−3*x*_ with *x* = 0 and 0.33. The capability for O_2_ evolution on LaMg_1/3_Ta_2/3_O_2_N can be attributed to the positive shift of VBM level, which demonstrates that the strategy of compositional tuning is effective. Although simultaneous H_2_ and O_2_ were observed for LaMg_1/3_Ta_2/3_O_2_N, two problems were found for this photocatalyst: One is the concurrent N_2_ evolution attributed to the self-oxidation of the photocatalyst; The other is the gradual decrease in the amount of accumulated O_2_, which indicates that the ORR proceeds in competition with water splitting. This suggests that the ORR takes place on the surface of bare LaMg_1/3_Ta_2/3_O_2_N because Rh_2−*y*_Cr_*y*_O_3_ is known to be selectively active for H^+^-reduction but not for the ORR [63]. However, the possibility that the ORR occurs on the surface of Rh_2−*y*_Cr_*y*_O_3_ cannot be denied for this case. This is because the cocatalyst loading conditions are slightly different for LaMg_1/3_Ta_2/3_O_2_N than for GaN:ZnO, and this could give rise to differences in cocatalyst structure and function.

We attempted to coat the entire surface of the photocatalyst particles with an amorphous oxyhydroxide layer to prevent the ORR. In this attempt, ORR is expected to be prevented although both of the cocatalyst and bare surface of photocatalyst were the active sites for ORR. Here we devised a novel surface coating material and method, which is the photodeposition of amorphous Ti-oxyhydroxide.

It is known that TiO_2_ dissolves in aqueous H_2_O_2_ solution by forming a Ti-peroxide complex [90]. This peroxide complex has an O(-I) state in the peroxo-ligand. The O(-I) state is an unusual valence state, which can be easily reduced or oxidized to O(-II) or O(0), respectively. Therefore, the peroxide complex can be easily reduced or oxidized by photoexcited electrons or holes, respectively, leading to the decomposition of peroxide to oxide on both of the cocatalyst and bare surface of photocatalyst. It is likely that the deposited material exists as an oxyhydroxide under the examined photodeposition conditions, but is more simply denoted as an oxide, such as TiO_2_.

In the procedure of TiO_2_ photodeposition, Ti peroxide solution is added to the photocatalytic reaction suspension, and TiO_2_ can be deposited *in situ* under bandgap excitation of the photocatalyst by a Xe lamp (300 W, *λ* ≥ 300 nm). During the photoirradiation, O_2_ evolution continues until all peroxide species were decomposed. After 1 day of photoirradiation, the O_2_ evolution terminates, and this can be regarded as the end of the photodeposition process. Then, the TiO_2_-deposited photocatalyst suspension is transferred to another reactor for high-pressure Hg lamp (450 W) irradiation, and the photocatalytic reaction is conducted therein.

**Figure 19.**
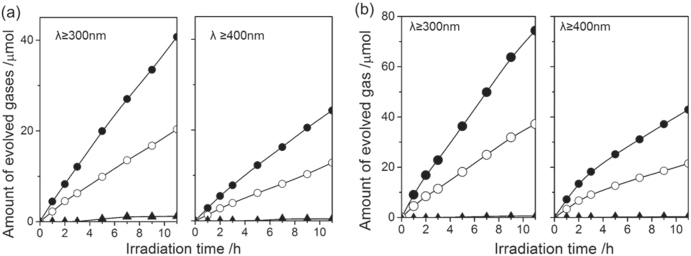
Gas evolution during water splitting on TiO_2_/Rh_*y*_Cr_2−*y*_O_3_/ LaMg_1/3_Ta_2/3_O_2_N(a) and on TiO_2_/SiO_2_/Rh_*y*_Cr_2−*y*_O_3_/LaMg_1/3_Ta_2/3_O_2_N(b) under UV+visible light irradiation and visible light irradiation alone. Catalyst 0.2 g, pure water 400 ml, inner-irradiation-type Pyrex reaction cell, with and without an aqueous NaNO_2_ solution filter, high-pressure mercury lamp (450 W). (●) H_2_, (○) O_2_, (▲) N_2_.

Figure 19(a) shows a typical time course of H_2_ and O_2_ evolution on TiO_2_/Rh_2−*y*_Cr_*y*_O_3_/LaMg_1/3_Ta_2/3_O_2_N. H_2_ and O_2_ evolve at a constant rate in a 2:1 ratio, the correct stoichiometry for water splitting. Notably, N_2_ evolution was almost completely prevented by the TiO_2_ coating. Covering the water oxidation sites with the deposited material improved the stability of the photocatalyst surface against self-oxidation, although the detail mechanism is unclear. This result clearly demonstrates that successful overall water splitting is possible after proper surface modification of the LaMg_1/3_Ta_2/3_O_2_N photocatalyst. Under either UV (*λ* ≥ 300 nm) or visible light (*λ* ≥ 400 nm) irradiation, overall water splitting proceeded. The photocatalytic activity of LaMg_1/3_Ta_2/3_O_2_N can be further enhanced by a double coating of TiO_2_/SiO_2_. Photodeposition of TiO_2_ on SiO_2_-precoated Rh_2−*y*_Cr_*y*_O_3_/LaMg_1/3_Ta_2/3_O_2_N resulted in approximately twice the photocatalytic activity (figure 19(b)). This SiO_2_ was coated not by photodeposition but by hydrolysis of tetraethylorthosilicate because no water-soluble Si-peroxide was available. With only SiO_2_ coating, N_2_ evolution cannot be suppressed. Therefore, the role of the SiO_2_-precoating was to improve the coating quality of the post-deposited TiO_2_.

**Figure 20.**
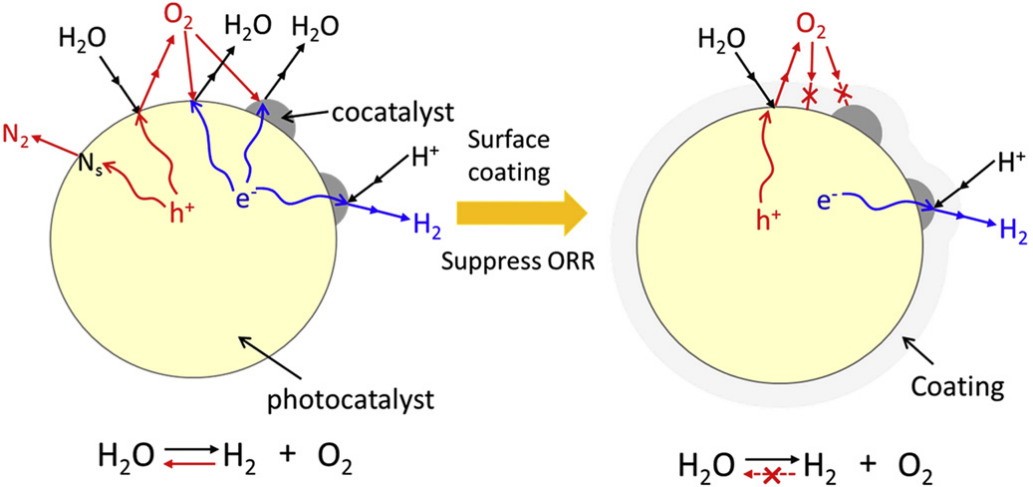
Schematic of the strategy for the surface coating of photocatalyst to achieve overall water splitting.

Scanning transmission electron microscope and energy dispersive x-ray spectroscopy analysis of the TiO_2_/SiO_2_-deposited photocatalyst revealed that the Ti and Si components were broadly dispersed on the surface of the photocatalyst particles, creating a core/shell-structured photocatalyst [87]. In this structure, both H_2_ and O_2_ evolution sites are covered with an overlayer, but water splitting is still possible. This indicates that the reactant and product can penetrate the coating layer. However, only the permeation of produced O_2_ from outside to inside of the coating layer can be suppressed, and this prevents the reverse reaction. The reaction mechanism for water splitting on the surface-coated photocatalyst is schematically summarized in the right part of figure 20. As far as we are aware, there has been no report indicating that such a model has been effective for overall water splitting.

This research demonstrates the effectiveness of controlling surface redox reactions in overall water splitting. The effective approaches demonstrated in this study should be extensible to various narrow bandgap photocatalysts for use in the development of more efficient photocatalyst systems.

## 8. Final remarks

In this review, various effective approaches to achieving photocatalytic overall water splitting were presented. A photocatalyst has two aspects, as it is both a semiconductor and a catalyst, and the design of water splitting photocatalysts should be carried out considering both of these aspects. The main focus of this review is to introduce work successfully demonstrating photocatalyst design based on this idea. Historical overview of this research subject is summarized in table 3. Since the first report of the Honda–Fujishima effect 40 years ago, the study of photocatalysts for water splitting has made remarkable progress due to the efforts of many researchers. Stable and steady water splitting with high QE was first made possible by the use of wide-gap semiconductors. Then, photocatalyst research shifted to visible light utilization. Several photocatalyst systems have been developed to achieve overall water splitting, including both direct and Z-scheme water splitting. Typically, GaN:ZnO solid solutions have a QE of a few percent at wavelengths of ca. 400–500 nm. Our recent development of the LaMg_1/3_Ta_2/3_O_2_N photocatalyst extends the usable wavelength range to 600 nm. However, the QE of LaMg_1/3_Ta_2/3_O_2_N for water splitting is about one-tenth that of GaN:ZnO. Therefore, the QE of the LaMg_1/3_Ta_2/3_O_2_N photocatalyst should be enhanced much further to enable better solar-to-hydrogen energy conversion efficiency. Our group has suggested that a tentative goal for research on hydrogen production via solar-driven overall water splitting using a particulate photocatalyst is to develop a stable material that can achieve a QE of 30% at 600 nm. In that case, the solar-to-hydrogen energy conversion efficiency is estimated to be ca. 5%. This stage may be a good starting point for examining practical applications.

**Table 3.** Historical overview of photocatalyst research.

Year	UV-light-sensitive photocatalysts	Reference	Year	Visible-light-sensitive photocatalysts	Reference
1972	Photoelectrochemical water splitting: Honda–Fujishima effect (TiO_2_ single crystal) Honda–Fujishima	[1]	1998	Valence band control: new oxide photocatalysts (BiVO_4_, SrTiO_3_:Rh, etc) Kudo	[5]
1980	Overall water splitting on particulate photocatalysts (TiO_2_, SrTiO_3_) Domen, Sato, Lehn	[8–10]	2000	Z-scheme water splitting (SrTiO_3_:Cr/Ta + WO_3_) Sayama	[91]
1986	New oxides with layered structures (K_4_Nb_6_O_17_, etc) Domen	[18]	2001	Non-oxides as new photocatalysts (sulfide, oxysulfide, nitride, oxynitride) Kudo, Domen	[5,6]
1992	New oxides with tunnel structures (BaTi_4_O_9_, etc) Inoue	[11]	2004	Z-scheme water splitting (SrTiO_3_:Rh + BiVO_4_) Kudo	[92]
1992	Effect of carbonate ion addition on promoting water splitting (TiO_2_, ZrO_2_, etc) Sayama	[13]	2005	Direct water splitting on d^10^-oxynitride(<480 nm) (GaN:ZnO) Inoue–Domen	[56]
1998	Highly active tantalate photocatalysts (NaTaO_3_, etc) Kudo	[14,18]	2013	Direct water splitting on d^0^-oxynitride(<520 nm) (ZrO_2_/TaON) Maeda-Domen	[93]
2000	d^10^-oxides as a new photocatalyst group (ZnGa_2_O_4_, Sr_2_SnO_4_, etc) Inoue	[21–27]	2014	Direct water splitting on metal-doped oxide(<520 nm) (SrTiO_3_:Rh/Sb) Kudo	[31]
2005	Overall water splitting on d^10^-nitride (Ge_3_N_4_) Inoue–Domen	[39]	2015	Direct water splitting on d^0^-oxynitride(<600 nm) (LaMg_1/3_Ta_2/3_O_2_N) Takata–Domen	[88]
